# Contact Region Estimation Based on a Vision-Based Tactile Sensor Using a Deformable Touchpad

**DOI:** 10.3390/s140405805

**Published:** 2014-03-25

**Authors:** Yuji Ito, Youngwoo Kim, Goro Obinata

**Affiliations:** 1 Graduate School of Engineering, Nagoya University, Furo-cho, Chikusa-ku, Nagoya 464-8603, Japan; 2 Daegu Research Center for Medical Devices and Green Engergy, Korea Institute of Machinery & Materials (KIMM), Deagu Techno Park R&D Center #1031, 711 Hosan-dong, Dalseo-gu 704-948, Korea; E-Mail: ywkim@kimm.re.kr; 3 EcoTopia Science Institute, Nagoya University, Furo-cho, Chikusa-ku, Nagoya 464-8603, Japan; E-Mail: obinata@mech.nagoya-u.ac.jp

**Keywords:** contact region estimation, flexible and conformable sensors and arrays, image processing, vision-based tactile sensors

## Abstract

A new method is proposed to estimate the contact region between a sensor and an object using a deformable tactile sensor. The sensor consists of a charge-coupled device (CCD) camera, light-emitting diode (LED) lights and a deformable touchpad. The sensor can obtain a variety of tactile information, such as the contact region, multi-axis contact force, slippage, shape, position and orientation of an object in contact with the touchpad. The proposed method is based on the movements of dots printed on the surface of the touchpad and classifies the contact state of dots into three types—A non-contacting dot, a sticking dot and a slipping dot. Considering the movements of the dots with noise and errors, equations are formulated to discriminate between the contacting dots and the non-contacting dots. A set of the contacting dots discriminated by the formulated equations can construct the contact region. Next, a method is developed to detect the dots in images of the surface of the touchpad captured by the CCD camera. A method to assign numbers to dots for calculating the displacements of the dots is also proposed. Finally, the proposed methods are validated by experimental results.

## 1. Introduction

Tactile receptors in the skin allow humans to sense multimodal tactile information such as the contact force, slippage, shape and temperature of a contacted object. By feeding back information from tactile receptors, humans can control their muscles dexterously. Therefore, tactile sensing is a crucial factor for robots to imitate skilled human behaviors. In consideration of practical applications, tactile sensors should meet three specific requirements. Firstly, flexible sensor surfaces are desirable because sensors should fit the object geometrically to avoid the contacted object from collapsing and enhance stability of the contact. Secondly, a simple structure is required for compactness of robots. Thirdly, for achieving dexterous and multifunctional robots, we need a sensor which can obtain various types of tactile information simultaneously.

Many types of tactile sensors have been developed using various sensing elements such as resistive, capacitive, piezoelectric, ultrasonic or electromagnetic devices [1,2]. In order to estimate the slippage of a contacted object, a sensor with array of strain gauges embedded in an elastic body has been proposed [3]. Standing cantilevers and piezo resistors arrayed in an elastic body have been developed for detecting shear stress [4]. Schmitz *et al.* have implemented twelve capacitance-to-digital converter (CDC) chips in a robot finger, providing twelve 16-bit measurements of capacitance [5]. Sensing elements based on a capacitive method have been arrayed on conductive rubber at regular intervals for measuring three components of stress [6].

However, the crucial practical issues remain unresolved. The structures of these sensors are complex and cannot satisfy the second requirement as described in the previous paragraph because theses sensors require many sensing elements and complicated wiring. Although a wire-free tactile sensor using transmitters/receivers [7] and a sensor using micro coils changing impedance by contact force [8] have been proposed, they are also packed in complex structures. Small sensors using microelectromechanical system (MEMS) have been manufactured [9–12]. However, the surfaces of these sensors are minimally deformable and cannot satisfy the first requirement as described above.

Differently to these sensors, vision-based sensors are suitable for tactile sensing [13–15]. Typical vision-based sensors can satisfy the first and second requirements as described above because they consist of the following two components: a deformable contact surface made of elastic material to fit its shape to contacted objects; and a camera to observe the deformation of the contact surface. Since multiple sensing elements and complex wiring are not required, compact vision-based sensors can be easily fabricated. Analysis of the deformation of the surface yields multiple types of tactile information. The two-layered dot markers embedded in the elastic body of the sensors have visualized the three-dimensional deformation of the elastic body to measure a three-axis contact force [16,17]. The markers are observed by a charge-coupled device (CCD) camera. The sensors consisting of rubber sheets with nubs, a transparent acrylic plate, a light source and a CCD camera have been developed [18,19]. Light traveling through the transparent plate is diffusely reflected at which the nubs contact the plate. The intensity of the reflected light captured by the CCD camera is transformed into the three-axis contact force. The sensor reported in [20] has estimated the orientation of an object by using the four corner positions of the reflector chips embedded in the deformable surface of the sensor. However, these sensors cannot satisfy the third requirement because they only detect single type of tactile information.

Moreover, although the sensors in the literature have provided information such as the contact force, slippage and shape of an object, the contact region between the sensor and an object also gives crucial information. The contact region allows us to estimate the shapes of objects in an accurate manner when combined with shape information from a sensor surface. Since geometric fit between a sensor and object occurs only in the contact region, the accurate estimation of an object's shape requires information about not only the sensor's shape but also the contact region. In consideration of implementing the sensor in robot hands, when the contact region is small, the grasped object can be easily rotated because the feasible contact moment is also small. In order to avoid the risk that the sensor surface tears or a grasped object collapses, and to enhance a grasping task's stability with a sufficiently large contact area, it is necessary to evaluate the contact pressure based on the contact region.

Among many sensors to sense contact forces, some sensors to measure force pressure distribution may detect the contact region based on the distribution [16–19]. In ideal situations, the force pressure becomes zero outside the contact region and does not become zero in the contact region. However, this assumption is violated by the stiffness of the elastic body. Moreover, in order to measure the pressure distribution, the sensors requires many arrayed sensing elements and wiring as described in above sentences. Some sensors have been proposed for obtaining the contact region directly. A sensor using regularly-arrayed cantilevers has been developed for estimating the contact region of objects from the deformation of the cantilevers in the elastic body [21]. However, this sensor also requires many arrayed sensing elements and the measurement error depends on the direction and position of the cantilevers. A large error can be caused when the cantilevers is far away from the contacted object. A finger-shaped tactile sensor based on optical phenomena has been developed for detecting the contact location [22]. The light travels from optical fibers into a hemispherical optical waveguide in the elastic sensor surface. When the sensor surface contacts the internal optical waveguide due to the contact between the sensor surface and the objects, light is reflected in the contact region. A position sensitive detector (PSD) receives the reflected light and thus the contact region is detected from the signals of the PSD. However, the surfaces of these sensors cannot fit with in the objects geometrically because the surfaces has elasticity but the inside optical waveguide is not deformed. Therefore, large contact region cannot be generated and that leads unstable contact.

We have proposed a vision-based tactile sensor that can sense multiple types of tactile information simultaneously including the slippage [23,24], contact region [25], shape [26], multi-axis contact force [27], position [25] and orientation [25] of an object. We have applied this sensor to prevent the object from slipping [28]. The sensor consists of a CCD camera, light-emitting diode (LED) lights and a hemispherical elastic touchpad for contacting the object. Because of the simple structure of the sensor and the deformable touchpad, our proposed sensor can satisfy the above three requirements: a deformable surface for the sensor; simple structure; and simultaneous acquisition of various types of tactile information. However, the previous method used for estimating the contact region required the strict restriction that the contact surface of the object must be flat or convex [25].

The purpose of this study is to estimate the contact region between the sensor and a contacted object without strict assumptions. A new proposed method is based on the movements of dots printed on the surface of the sensor. The contact state of the dots is classified into three types—the non-contacting dot, the sticking dot and the slipping dot. Considering the movements of the dots, equations are formulated to discriminate between the contacting dots and the non-contacting dots and modified by selecting the appropriate time interval and introducing the threshold values. A set of the contacting dots discriminated by the formulated equations can construct the contact region. Next, an image processing method is proposed to detect the dots in images of the surface of the sensor captured by the CCD camera. A method to assign numbers to dots for calculating the displacements of the dots is also proposed. Finally, the methods proposed methods are validated by experimental results.

## 2. Vision-Based Tactile Sensor

**Figure 1.**
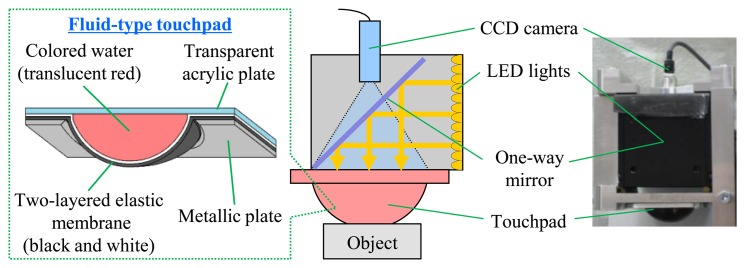
Configuration of the vision-based tactile sensor and the deformable touchpad.

**Figure 2.**
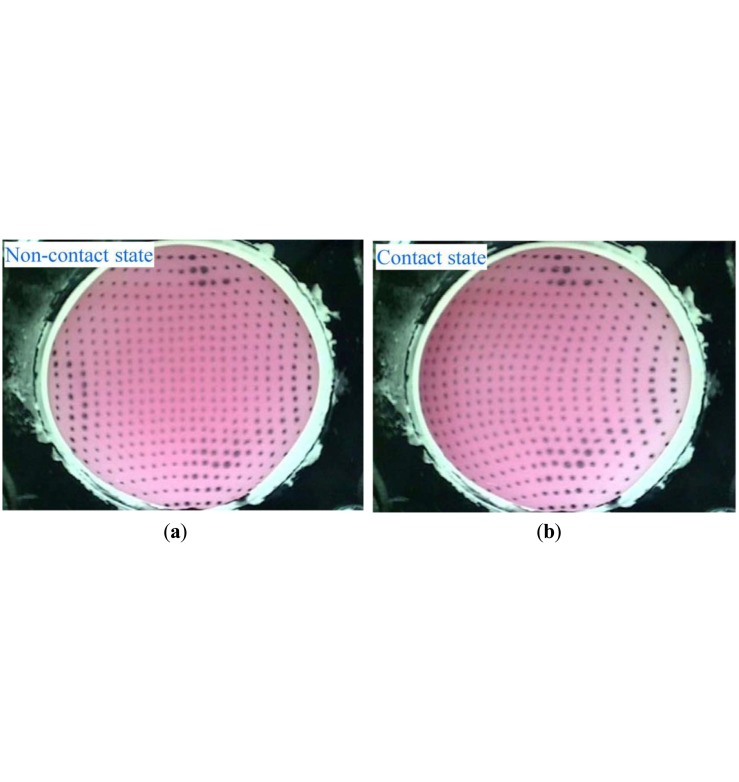
The image captured by the CCD camera when (**a**) the touchpad is not in contact with an object; and (**b**) when the touchpad contacts an object moving in the tangential direction.

Figure 1 shows the configuration of a vision-based tactile sensor which consists of a CCD camera, LED lights, and a deformable fluid-type touchpad. The dimensions of the LED lights and the CCD camera are 60 × 60 × 60 mm and 8 × 8 × 40 mm, respectively. The fluid-type touchpad is hemispherical, with a curvature radius and height of 20 mm and 13 mm. The surface of the touchpad is made of an elastic membrane constructed of silicon rubber; the inside of the membrane is filled with translucent, red-colored water. A dotted pattern is printed on the inside of the touchpad surface to observe the deformation of the touchpad. When the touchpad comes in contact with objects, analysis of the deformations yields multimodal tactile information, using an image of the inside of the deformed touchpad captured by the CCD camera. Figure 2 shows the captured images, sized 640 × 480 effective pixels, when the touchpad does not contact and contact an object, respectively. Our proposed sensor can obtain multiple types of tactile information, including the contact region, multi-axis contact force, slippage, shape, position and orientation of an object [23–27].

## 3. Estimation of Contact Region

### 3.1. Theory for Estimating the Contact Region

**Figure 3.**
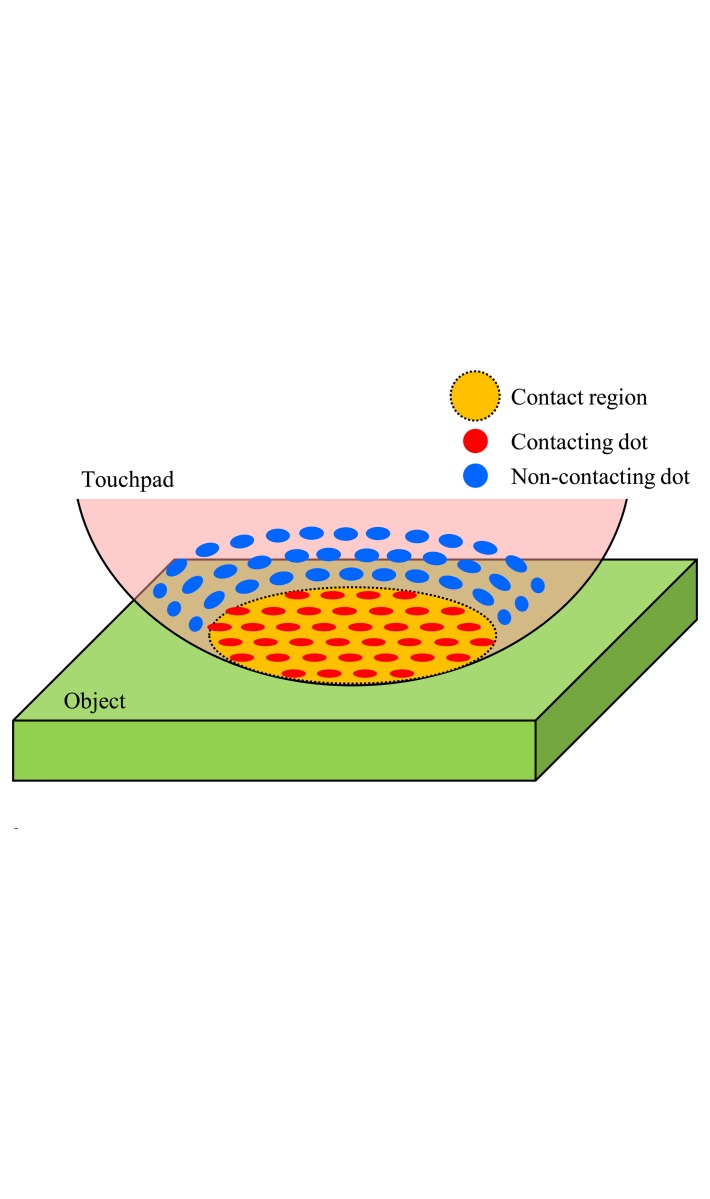
The contact region between the touchpad and an object is constructed as a set of the contacting dots.

In order to estimate the contact region between the touchpad and an object, we focus on the approach that the printed dot patterns on the surface of the touchpad can be considered to be sensing elements. If each dot contacting the object can be discriminated, the contact region can be constructed as a set of dots in the contact region as shown in Figure 3. Although the other sensors use many sensing devices and wiring for obtaining the contact region [16–19,21], our approach is advantageous because fewer sensing elements and less wiring is required, thereby generating a more compact size and structure. Moreover, the method is generalized because it can be applied to other sensors including dots/markers on the sensor surface. Differently to using many sensing devices, the size of dots can easily be produced in smaller sizes by the printing technique, and thus high resolution is expected. The sensor for obtaining the contact region in [22] cannot fit with in the objects geometrically because of the inside solid body. Our previous work in [25] required the strict restriction that the contact surface of the object must be flat or convex. Differently from these previous works, our sensor without many sensing devices can deform because of the elastic touchpad and does not require strict assumptions to objects, which are also advantageous. In the next section, we discriminate dots to construct the contact region.

### 3.2. Discrimination of Dots

The contact state of a dot is classified into three types—the non-contacting dot, the sticking dot and the slipping dot. The non-contacting dot is a dot that lies outside of the contact region. The sticking dot is defined as a dot that is in the contact region but does not slip, while the slipping dot slips on an object in the contact region. The contacting dots include the sticking dots and the slipping dots. In order to construct the contact region, the multiple types of dots are discriminated, and the contacting dots are extracted.

To solve the problem of discriminating among the dots, we focus on dynamic information concerning the dots. Considering the movements of the dots with reference to an object, we formulate equations to discriminate between the contacting dots and the non-contacting dots. Here, calculation of the positions and movements of the dots is described in the next section.

**Figure 4.**
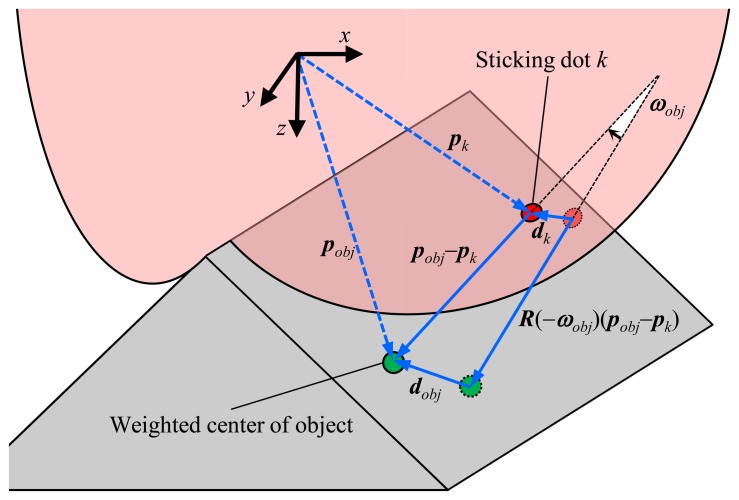
Geometric relationship between the object and the sticking dot *k*.

Firstly, we address the discrimination of the sticking dots. When a certain dot is in contact with the object without slippage, the movement of the dot is geometrically-determined from the movement of the object. The sticking dot must satisfy the following equation:

(1) $$d_{\text{obj}}=d_{k}-R(-\omega_{\text{obj}})(p_{\text{obj}}-p_{k})+(p_{\text{obj}}-p_{k})=d_{k}+\left\{I-R(-\omega_{\text{obj}})\right\}(p_{\text{obj}}-p_{k})$$

where ***ω****_obj_* is the rotation angle of the object defined as Euler angle, ***I*** is the identity matrix of size three; ***d****_obj_* and ***d****_k_* are the three-dimensional displacements of the object and a certain sticking dot *k*, respectively; ***p****_obj_* and ***p****_k_* are the three-dimensional positions of the object and the sticking dot *k*, respectively; and ***R***(−***ω****_obj_*) is the rotation (square) matrix of size three with reference to the three-dimensional angle −***ω****_obj_*. Here, the rotation angle, the displacement and the position of the object refer to the mean for the weighted center of the object. Figure 4 shows this geometric relationship between the object and the sticking dot *k* in Equation (1).

In order to apply Equation (1) to all dots, we must obtain the rotation angle, the displacement and the position of the object. Although the values cannot be directly obtained, our previous method can be used to estimate the rotation angle and the displacement of the object under the assumption that the at least a set of nine dots are does not slip on the surface of an object [25]. This assumption is achieved by preventing the object from slipping when we apply our previous method [28].

We introduce the contact reference dot previously proposed in [25] to calculate the rotation angle and the displacement of the object. From among the multiple dots printed on the surface of the touchpad, the contact reference dot *k_ref_*, is defined in the following equation as the dot with the greatest displacement from the initial position:

(2) $$k_{ref}=\underset{k}{\arg\max}\left|p_{k}\left(t_{c}\right)-p_{k}\left(t_{0}\right)\right|$$

where ***p****_k_* is the three-dimensional position of a dot *k*, and *t_c_* and *t*_0_ are the current time and the initial time at which the touchpad is not in contact with any objects, respectively.

The contact reference dot is defined such that it is always in contact with the object. Moreover, the contact reference dot does not slip on the surface of a contacted object, because our previous method can be applied to prevent the object from slipping [28]. As a result of this characteristic, the displacement and the rotation angle of the contact reference dot are equal to those of the object as demonstrated in an earlier manuscript [25]. Therefore, the contact reference dot always satisfies the following equations based on Equation (1):

(3) $$\left\{ \begin{array}{l} \omega_{\text{obj}}=\omega_{\text{ref}}\\ d_{\mathrm{obj}}=d_{\mathrm{ref}}+\left\{I-R\left(-\omega_{\text{obj}}\right)\right\}\left(p_{\text{obj}}-p_{\text{ref}}\right)=d_{\mathrm{ref}}+\left\{I-R(-\omega_{\text{ref}})\right\}\left(p_{\text{obj}}-p_{\text{ref}}\right) \end{array}\right.$$

where ***ω****_ref_*, ***d****_ref_* and ***p****_ref_* are the rotation angle, the displacement and the position of the contact reference dot *k_ref_*.

Next, we calculate the position of the object. When ***ω****_obj_* = ***ω****_ref_* = ***0*** without rotation, ***p****_obj_* is eliminated in Equations (1) and (3) because ***R***(–***ω****_obj_*) = ***R***(–***ω****_ref_*) = ***I***. When ***ω****_ref_* is not equal to zero, ***p****_obj_* is given as follows by transforming Equation (3):

(4) $$p_{\text{obj}}={\left\{I-R(-\omega_{\mathrm{ref}})\right\}}^{-1}\left(d_{\text{obj}}-d_{\text{ref}}\right)+p_{\text{ref}}$$

Therefore, substituting Equation (4) into Equation (1) can eliminate the position of the object ***p****_obj_* as follows:

(5) $$d_{\text{obj}}=\{\begin{array}{ll} d_{k} & \left(R\left(-\omega_{\mathrm{ref}}\right)=I\right)\\ d_{k}+\left\{I-R\left(-\omega_{\text{obj}}\right)\right\}\left({\left\{I-R\left(-\omega_{\text{ref}}\right)\right\}}^{-1}\left(d_{\text{obj}}-d_{\text{ref}}\right)+p_{\text{ref}}-p_{k}\right) & \left(R\left(-\omega_{\text{ref}}\right)\neq I\right) \end{array}$$

Moreover, substituting ***ω****_obj_* = ***ω****_ref_* from Equation (3) into Equation (5) eliminates the rotation angle of the object ***ω****_obj_* as follows:

(6) $$d_{\text{obj}}=\{\begin{array}{ll} d_{k} & \left(R\left(-\omega_{\text{ref}}\right)=I\right)\\ d_{k}+\left(d_{\text{obj}}-d_{\text{ref}}\right)+\left\{I-R\left(-\omega_{\text{ref}}\right)\right\}\left(p_{\text{ref}}-p_{k}\right) & \left(R\left(-\omega_{\text{ref}}\right)\neq I\right) \end{array}$$

Note that ***d****_obj_* = ***d****_ref_* when ***R***(-***ω****_ref_*) = ***I*** without rotation from Equation (3). Therefore, we can simplify Equation (6) into the following equation:

(7) $$d_{\text{ref}}=d_{k}+\left\{I-R\left(-\omega_{\text{ref}}\right)\right\}\left(p_{\text{ref}}-p_{k}\right)$$

Consequently, a sticking dot can be discriminated by considering the relative displacement between the dot and the contact reference dot based on Equation (7).

Secondly, the discrimination of non-contacting dots is considered. When a dot is not in contact with the object, the dot does not satisfy Equation (7) as follows:

(8) $$d_{\text{ref}}\neq d_{k}+\left\{I-R\left(-\omega_{\text{ref}}\right)\right\}\left(p_{\text{ref}}-p_{k}\right)$$

Thirdly, the difficult problem of the discrimination of slipping dots is considered—the displacements of the slipping dots are not equal to the displacement of the object because of the slippage. The approach used in this paper to solve this problem is to use the normal component of the displacement, which is perpendicular to the surface of the object. Although the slipping dot slips on the surface of the object, the dot does not move in the normal direction that is perpendicular to the surface of the object. Therefore, the normal component of the displacement of the dot is independent of the slippage. The normal component of the displacement of the slipping dot is equal to that of the object as follows:

(9) $$d_{\text{ref}}\cdot n_{k}=\left\lfloor d_{k}+\left\{I-R\left(-\omega_{\text{ref}}\right)\right\}\left(p_{\text{ref}}-p_{k}\right)\right\rfloor \cdot n_{k}$$

where ***n****_k_* is the unit vector perpendicular to the surface of the object around the position of a slipping dot *k*. The value of ***n****_k_* is calculated by using the three-dimensional shape of the touchpad based on the method published in a previous manuscript [26].

We have formulated the conditions Equations (7)–(9) for discriminating among sticking dots, non-contacting dots, and slipping dots. In fact, the estimation of the contact region only requires the discrimination between the non-contacting dots and the contacting dots. However, the non-contacting dots may satisfy Equations (8) and (9) simultaneously. In order to avoid this problem, Equations (7) and (9) are applied to discriminate among the dots depending on the previous (one sampling step earlier) contact state of the dots. When a dot was a non-contacting dot in the previous state, we apply Equation (7) to determine the current contact state. The dot satisfying Equation (7) is regarded as the contacting dot. Otherwise, it is regarded as a non-contacting dot. When a dot was the contacting dot in the previous state, Equation (9) is applied. The dot satisfying Equation (9) is regarded as a contacting dot. Otherwise, it is regarded as a non-contacting dot. In the following section, the three-dimensional positions and displacements of the dots are calculated, and Equations (7) and (9) are modified for accuracy.

#### 3.3. Modification of Equations Discriminating among the Dots

In order to calculate the three-dimensional positions of dots, the three-dimensional shape of the surface of the touchpad is used, which is estimated in our previous research reported in [26]. Images captured by the CCD camera contain the two-dimensional positions of the dots. By combining the three-dimensional shape of the surface of the touchpad with the two-dimensional positions of the dots in the images, the three-dimensional positions of the dots are calculated based on the geometric relationship described in [25].

Next, the displacements of the dots are calculated as the changes in positions from the previous time *t_p_* to the current time *t_c_*. Here, the value for the previous time *t_p_* must be selected appropriately because a large passage of time decreases the responsiveness of the proposed method. Moreover, the contact state of a dot may change between the previous time *t_p_* to the current time *t_c_* when *t_c_*–*t_p_* and the displacement of the dot are significantly large. Conversely, if the displacement of the dot is too small because of small *t_c_*–*t_p_*, the value is inappropriate, because it is significantly influenced by noise and the error of estimating the positions of the dots. In order to address this compromise, we introduced a threshold value *D*_max_ for determining *t_p_* as given in the following equation:

(10) $$t_{p}=\max\left(t\right)s.t.D_{\max}<\max\left|p_{k}\left(t_{c}\right)-p_{k}\left(t_{p}\right)\right|\left(k\in K\right)$$

where *K* is a set of numbers of all dots and Equation (10) indicates that the previous time *t_p_* is determined such that the displacement of the dot is large enough, and a recent time is selected for making *t_c_*–*t_p_* smaller. Finally, the displacements of the dots in Equations (7) and (9) are defined as follows:

(11) $$\left\{ \begin{array}{l} d_{\text{ref}}=p_{\text{ref}}\left(t_{c}\right)-p_{\text{ref}}\left(t_{p}\right)\\ d_{k}=p_{k}\left(t_{c}\right)-p_{k}\left(t_{p}\right) \end{array}\right.$$

Here, Equations (7) and (9) cannot be directly applied because the calculated positions of dots include the estimation error due to the shape-sensing error [26] and noise in the captured image. Therefore, Equation (7) is modified to decrease the effects of the estimation error of the positions as follows:

(12) $$\left|d_{\text{ref}}\left(t_{p},t_{c}\right)-\left\{d_{k}\left(t_{p},t_{c}\right)+\left\{I-R\left(-\omega_{\text{ref}}\left(t_{p},t_{c}\right)\right)\right\}\left(p_{\text{ref}}\left(t_{c}\right)-p_{k}\left(t_{c}\right)\right)\right\}\right|<\delta_{d}\frac{d_{\max}}{D_{\max}}$$

where ***ω****_obj_*(*t_c_*, *t_p_*) is the rotation angle of the object from the previous time *t_p_* to the current time *t_c_*. ***d****_obj_*(*t_c_*, *t_p_*) and ***d****_k_*(*t_c_*, *t_p_*) are the displacements of the contact reference dot and the dot *k* from the previous time *t_p_* to the current time *t_c_*. ***δ****_d_* is a threshold value, and *d*_max_ is defined as follows:

(13) $$\begin{array}{cc} d_{\max}=\max\left|p_{k}\left(t_{c}\right)-p_{k}\left(t_{p}\right)\right| & \left(k\in K\right) \end{array}$$

Here, *d*_max_/*D*_max_ in Equation (12) can normalize the sensitivity of Equation (12) depending on the small difference between max|***p****_k_*(*t_c_*)-***p****_k_*(*t_p_*)| (= *d*_max_) and *D*_max_ in Equation (10), when the previous time *t_p_* is selected. We also modify Equation (9) as follows:

(14) $$\left|\begin{array}{l} d_{\text{ref}}\left(t_{p},t_{c}\right)\cdot n_{k}\left(t_{c}\right)\\ -\left\{d_{k}\left(t_{p},t_{c}\right)+\left\{I-R\left(-\omega_{\text{ref}}\left(t_{p},t_{c}\right)\right)\right\}\left(p_{\text{ref}}\left(t_{c}\right)-p_{k}\left(t_{c}\right)\right)\right\}\cdot n_{k}\left(t_{c}\right) \end{array}\right|<\delta_{d}\frac{d_{\max}}{D_{\max}}$$

Here, ***n****_k_*(*t_c_*) is the unit vector perpendicular to the surface of the object around the position of a slipping dot *k* at *t_c_*. By applying Equations (12) and (14) to the dots, we discriminate between the non-contacting dots and the contacting dots.

Here, although we consider distinguishing slipping dots temporarily to discriminate the contacting dot appropriately, the estimation of the contact region finally requires only the discrimination between contacting dots and non-contacting dots. It is not required to consider slipping dots directly because the only Equations (12) and (14) can conclude the discrimination.

#### 3.4. Image Processing for Detecting the Dots in Captured Images

**Figure 5.**
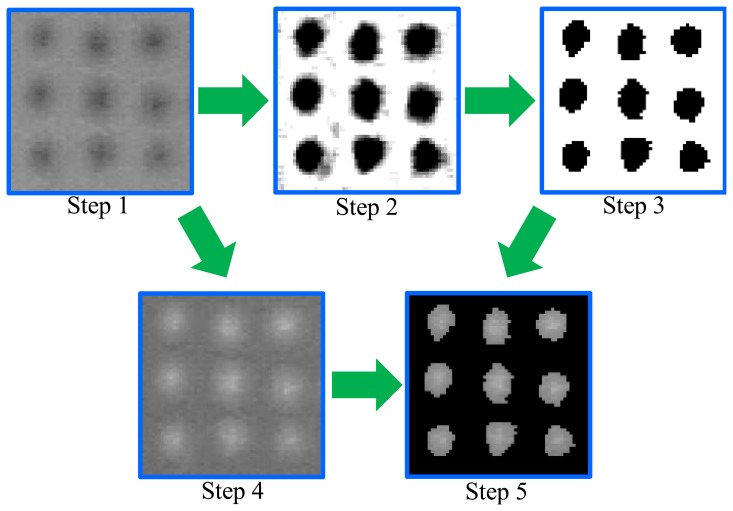
Image processing to detect the dots in captured images.

In the previous sections, we have proposed a method using the two-dimensional positions of the dots in images captured by the CCD camera for calculating the three-dimensional positions. Therefore, accuracy of the proposed method depends on the detection accuracy of the dots in the images. An image processing method is proposed to detect the dots accurately. The following six steps yield the positions of the dots as shown in Figure 5.

Step 1: The captured color image is transformed into a gray scale image.
Step 2: The contrast in the gray scale image is emphasized.
Step 3: The regions of the dots are extracted by binarizing the emphasized image. The extracted regions are represented by the black color in Figure 5.
Step 4: The brightness of the gray scale image is inverted.
Step 5: The brightness of the inverted image is extracted in the regions of the dots
Step 6: The position of each dot is obtained by calculating the brightness center in the region based on 0- and 1st-order moments as follows:
  (15) $$\left(x_{k},y_{k}\right)=\left(\frac{m_{k,1,0}}{m_{k,0,0}},\frac{m_{k,0,1}}{m_{k,0,0}}\right)$$
  where (*x_k_*, *y_k_*) are the *x*- and *y*-directional positions of the dot *k* in the image. The *v*- and *w*-order moments are defined as follows:
  (16) $$\begin{array}{cc} m_{k,v,w}=\underset{i}{\text{∑}}\underset{j}{\text{∑}}I\left(i,j\right)i^{v}j^{w} & \left(\left(i,j\right)\in R_{k}\right) \end{array}$$
  where *I*(*i*, *j*) is the inverted brightness of the pixel (*i*, *j*), and *R_k_* is the region of the dot *k* extracted in Step 3. Here, inverting the brightness of the image in Step 3 can increase the detection accuracy of the dots. Note that the border of the extracted region of each dot may contain the error because of binarizing the image. If the brightness of the image is not inverted, the brightness at the border of the region is larger than the brightness at the center of the region. Therefore, the brightness of the border is dominant and thus decreases accuracy.

#### 3.5. Numbering the Detected Dots

By the method introduced in the previous section, the positions of the dots have been obtained. However, the data are insufficient for calculating the displacements of the dots. In order to obtain the displacements, the identical dots must be identified between two different images. One approach is tracking the position of each dot by starting from the previous position. However, when the dots move quickly and the displacements of the dots are large, as shown in the difference between Figure 2, tracking methods may not be successfully applied, and thus a different dot is identified.

**Figure 6.**
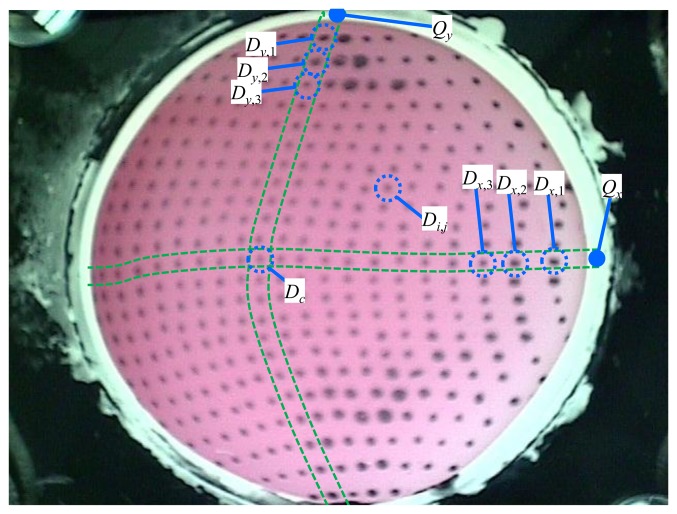
The process of assigning numbers to all dots in the image.

As an alternative to the tracking methods, the approach applied in this paper is to assign an identification number to each dot. When each dot has a fixed number, it can be easily identified even if it moves quickly. The following five steps are applied to assign numbers to the dots as shown in Figure 6:

Step 1: The constant points, *Q_x_* and *Q_y_*, were defined.
Step 2: The dots *D_x_*_,1_ and *D_y_*_,1_ which are the dots nearest to *Q_x_* and *Q_y_*, respectively, are selected.
Step 3: The dots *D_x_*_,_*_k_* and *D_y_*_,_*_k_* are located starting from *D_x_*_,1_ and *D_y_*_,1_, respectively, in order of increasing *k*. Here, *D_x_*_,_*_k_* and *D_y_*_,_*_k_* are found near the positions ***P****'_x_*_,_*_k_* and ***P****'_y_*_,_*_k_*, respectively, which predict the positions of *D_x_*_,_*_k_* and *D_y_*_,_*_k_*. Because the distances between two dots should be gradually changed when the next dots are searched, the following approximations are satisfied:
  (17) $$\begin{array}{c} P_{x,k}-P_{x,k-1}≅P_{x,k-1}-P_{x,k-2}\\ P_{y,k}-P_{y,k-1}≅P_{y,k-1}-P_{y,k-2} \end{array}$$
  where ***P****_x_*_,_*_k_* and ***P****_y_*_,_*_k_* are the positions of *D_x_*_,_*_k_* and *D_y_*_,_*_k_*, respectively. Therefore, the positions ***P****'_x_*_,_*_k_* and ***P****'_y_*_,_*_k_* predicting ***P****_x_*_,_*_k_* and ***P****_y_*_,_*_k_* are defined as follows:
  (18) $$\begin{array}{c} P{'}_{x,k}=P_{x,k-1}+\left(P_{x,k-1}-P_{x,k-2}\right)\\ P{'}_{y,k}=P_{y,k-1}+\left(P_{y,k-1}-P_{y,k-2}\right) \end{array}$$
  ***P****'_x_*_,_*_k_* and ***P****'_y_*_,_*_k_* can predict ***P****_x_*_,_*_k_* and ***P****_y_*_,_*_k_* accurately even if the surface of the touchpad is significantly deformed because of the relationships defined in Equation (17). When *k* is 2, a constant vector is applied instead of (***P****_x_*_,_*_k_*_−1_−***P****_x_*_,_*_k_*_−2_) and (***P****_y_*_,_*_k_*_−1_−***P****_y_*_,_*_k_*_−2_) in Equation (18).
Step 4: The central dot *D_c_* is identified, which corresponds to *D_x_*_,_*_v_* and *D_y,w_* detected in Step 3, where *v* and *w* are arbitrary. There is only one dot defined as *D_c_* in the image. The central dot *D_c_* is assigned the value (12,12).
Step 5: The dot with the number *i*, *j* that is the *i*-th dot from the left and the *j*-th dot from the top is defined. When starting from *D_c_* (12,12), each dot *i*, *j* is searched by using the predicted position ***P****'_i_*_,_*_j_* which is determined based on the relationship in Equation (17) as follows:
  $$P{'}_{i,j}=\left\{ \begin{array}{l} P_{i,j\pm 1}+\left(P_{i,j\pm 1}-P_{i,j\pm 2}\right)\\ P_{i\pm 1,j}+\left(P_{i\pm 1,j}-P_{i\pm 2,j}\right)\\ P_{i\pm 1,j\pm 1}+\left(P_{i\pm 1,j}-P_{i\pm 1,j\pm 1}\right)+\left(P_{i,j\pm 1}-P_{i\pm 1,j\pm 1}\right) \end{array}\right.$$

The equations are used to predict ***P****_i_*_,_*_j_*. In these steps, numbers (*I* = 1,2,…,23, *j* = 1,2,…,23) can be assigned to all dots in each image.

### 4. Experimental Results

**Figure 7.**
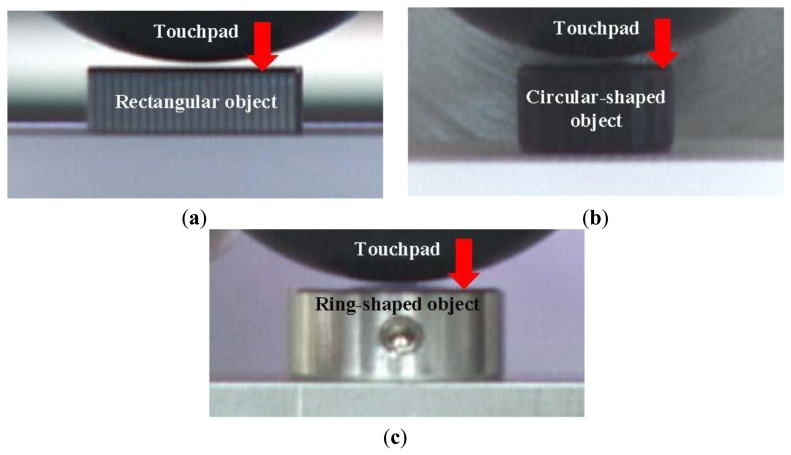
The side view images when (**a**) the touchpad was in contact with a rectangular object; (**b**) the touchpad was in contact with a circular-shaped object; and (**c**) the touchpad was in contact with a ring-shaped object.

In this section, the proposed method is confirmed by the experimental results. The proposed sensor was fixed on a movable stage, in contact with variously-shaped objects such as a rectangular object, a circular-shaped object and a ring-shaped object as shown in Figure 7. When the object was moved in the normal direction, the contact region was calculated by using the proposed method. The actual shapes of the contact regions were observed from the image of the inside of the touchpad when the objects were enough deeply contacted. We set the parameters *D*_max_ and ***δ****_d_* to 12.0 pixel and 2.5 pixel, respectively, according to trial and error.

We evaluate the proposed method by considering the results of discriminated dots in the experiment. In the following figures, true positive dots are correctly discriminated contacting dots. On the other hand, false positive dots are non-contacting dots really but are discriminated as the contacting dots. False negative dots are contacting dots really but are discriminated as the non-contacting dots. Therefore, when the proposed method are successfully applied, there are few false positive/negative dots.

**Figure 8.**
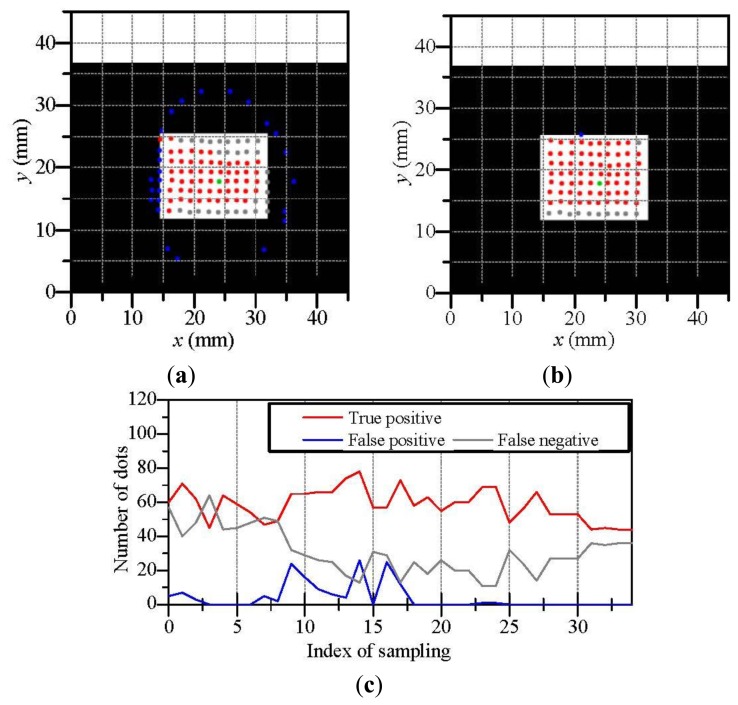
The estimation result of contact region when the touchpad contacted a rectangular object; (**a**) is a worse case; (**b**) is a better case; (**c**) is the variation of the estimation when the contact became increasingly deeper.

Figure 8 shows the results of the estimated contact region when the touchpad contacted a rectangular object. Although a worse case in Figure 8a includes the some false positive/negative dots, there are few false positive/negative dots in a better case in Figure 8b. Figure 8c shows the variation of the numbers of true positive, false positive and false negative dots. Here, the touchpad was moved such that the contact of the object became increasingly deeper with an increase in the index of sampling. The contact became enough deep for estimating the contact region after the index of sampling became approximately 10. In the experiment for the rectangular object, false positive dots were hardly occurred whereas there were some false negative dots. It seems that the three-dimensional dot positions based on the touchpad shape contain positional errors because the estimation accuracy of the touchpad shape by the previous work [26] can degrade around the sharp edge of the rectangular object, where the touchpad shape is used for calculating dot positions. However, we can see that the set of the contacting dots in Figure 8b construct a rectangular shape approximately. We regard the shape of the contact region as a set of contacting dots which constructing the contact region.

**Figure 9.**
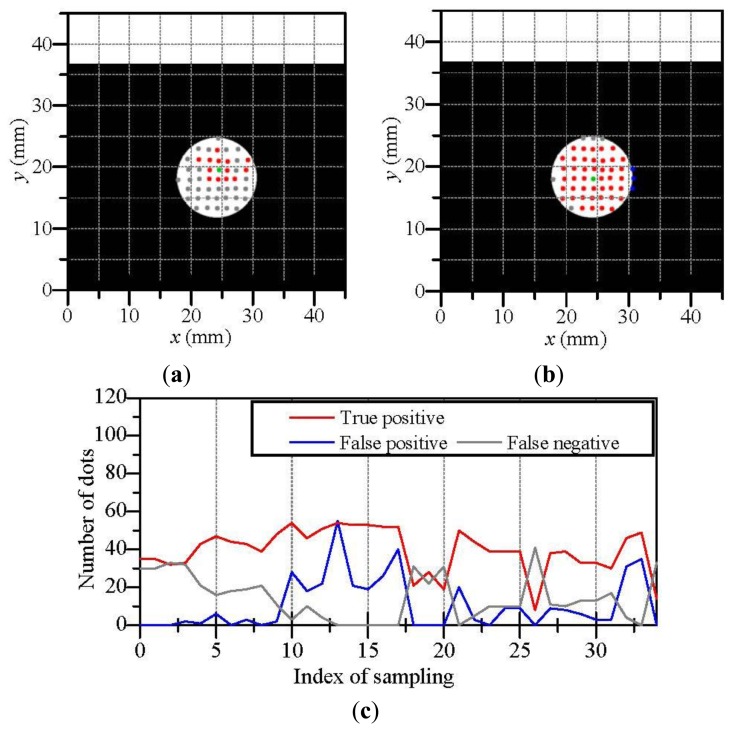
The estimation result of contact region when the touchpad contacted a cylinder-shaped object; (**a**) is a worse case; (**b**) is a better case; (**c**) is the variation of the estimation when the contact became increasingly deeper.

Next, Figure 9 illustrates the results of the estimated contact region when the touchpad contacted a cylinder-shaped object. Figure 9a,b shows a worse case and a better case, respectively. Figure 9c shows the variation of the numbers of true positive, false positive and false negative dots. The contact of the object became increasingly deeper with an increase in the index of sampling and the contact became enough deep for estimating the contact region after the index of sampling became approximately 10. We can see that the set of the contacting dots in the better case construct a circular shape whereas there are some worse cases as shown in Figure 9c. It seems that the estimation error of the dot positions also invokes false positive/negative dots.

**Figure 10.**
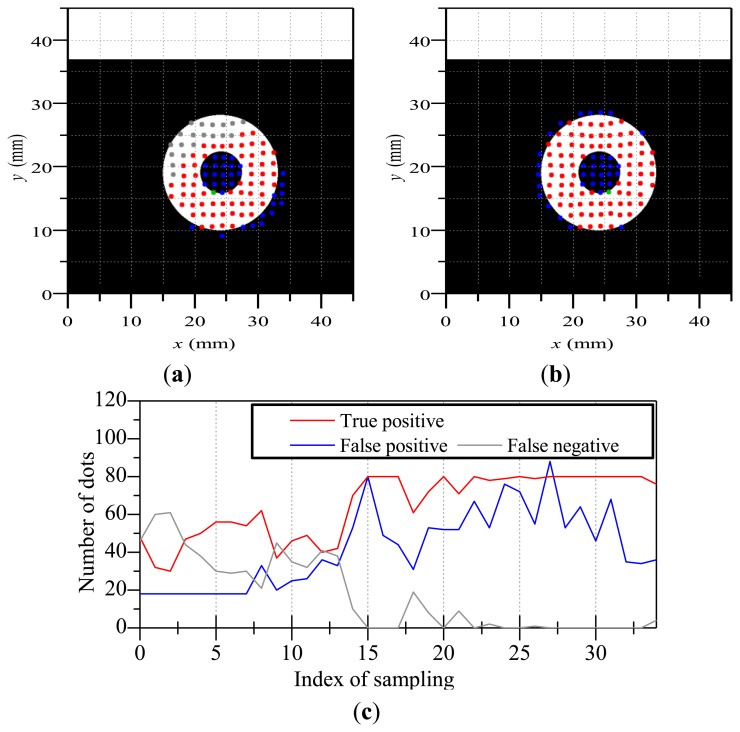
The estimation result of contact region when the touchpad contacted a ring-shaped object; (**a**) is a worse case; (**b**) is a better case; (**c**) is the variation of the estimation when the contact became increasingly deeper.

Finally, Figure 10 illustrates the results of the estimated contact region when the touchpad contacted a ring-shaped object. Figure 10a,b shows a worse case and a better case, respectively. Figure 10c shows the variation of the numbers of true positive, false positive and false negative dots. The contact of the object became increasingly deeper with an increase in the index of sampling and the contact became enough deep for estimating the contact region after the index of sampling became approximately 15. In the case of the ring-shaped object, false positive dots were occurred on the inside of the ring. This is because that the membrane on the inside of the ring can move along with the ring-shaped object due to the stiffness of the membrane. However, many dots were appropriately discriminated except the inside of the ring. When we use a softer/thinner membrane for the surface of the touchpad, false positive dots will be diminished on the inside of the ring.

In these results of Figures 8, 9 and 10, we have observed some errors of false positive/negative dots. These errors are due to the estimation error of the three-dimensional positions of dots which are invoked by the estimation error of the touchpad shape [26] and the estimation error of the two-dimensional dot position in images. However, it seems that the these errors can be decrease by using a camera with higher resolution and higher signal-noise ratio and by painting the dot pattern with higher accuracy, where the dot pattern is currently painted by hand work. Moreover, the sets of the contacting dots can construct the shape of the actual contact region in some cases. Therefore, we consider that the proposed method can be applied for estimating the contact region and the estimation accuracy of the proposed method will be increased by improving the hardware such as a camera and a dot pattern.

### 5. Conclusions and Discussion

We have proposed a new method to estimate the contact region between a touchpad and a contacted object without strict assumptions. The proposed method is based on the movements of dots printed on the surface of the touchpad. The contact state of the dots has been defined as three types—the non-contacting dot, the sticking dot and the slipping dot. In consideration of the movements of the dots, equations have been formulated to discriminate between the contacting dots and the non-contacting dots. The equations have been modified to decrease the effects of noise and the error of estimating the positions of the dots. A set of the contacting dots discriminated by the formulated equations can construct the contact region. Next, a six-step image processing has been also proposed to detect the dots in captured images. Next, a method has been developed to assign numbers to dots for calculating their displacements. Finally, the validation of our proposed methods has been confirmed by experimental results.

Although some errors of false positive/negative dots remained in the experimental results, more accurate discrimination of the dots will be expected by enhancing the calculation accuracy of the three-dimensional positions of dots. It seems that the better estimation of the positions can be achieved by using a camera with higher resolution and higher signal-noise ratio and by painting the dot pattern with higher accuracy, where the dot pattern is currently painted by hand work.

The method can be generalized because it can be applied to other sensors including dots/markers on the sensor surface. The size of each dot of the developed sensor in this research is relatively large because the dot pattern is painted by hand work. However, differently to using many sensing elements, the size of the dots can easily be made smaller by the printing technique, and thus high resolution is expected. The proposed sensor can be fabricated easily at minimal cost, because it has a simple structure and does not require complex sensing elements or wiring. Although the size of the sensor developed and used in this research is relatively large, it can be easily downsized by using a smaller CCD or complementary metal-oxide semiconductor (CMOS) camera with high resolution.

In the process of contributing to this paper, our vision-based sensor has been developed to a greater level of practicality. Combined with our previous research [23–27], we believe that the vision-based sensor can simultaneously obtain multiple types of tactile information, including the contact region, multi-axis contact force, slippage, shape, position and orientation of an object in contact with the touchpad. Future investigation will include the implementation of fluid-type tactile sensors in industrial and medical applications, and in various practical applications, such as for robot hands for dexterous handling.
